# Hyperuricemia-Informed Survival Machine-Learning Prediction of Post-Thrombotic Syndrome After Unprovoked DVT: A Dual-Center Prospective Study

**DOI:** 10.3390/diagnostics16010088

**Published:** 2025-12-26

**Authors:** Yajing Li, Hongru Deng, Yongquan Gu

**Affiliations:** 1 Department of Vascular Surgery, Xuanwu Hospital, Capital Medical University, Beijing 100053, China; 2 Department of Vascular Surgery, Fu Xing Hospital, Capital Medical University (FXH-CMU), Beijing 100038, China

**Keywords:** post-thrombotic syndrome, unprovoked DVT, survival machine learning, hyperuricemia, decision-curve analysis

## Abstract

**Background/Objectives**: Post-thrombotic syndrome (PTS) following unprovoked deep vein thrombosis (DVT) lacks readily available, calibrated risk estimates at defined follow-up horizons. Building on signals that thrombus burden, care processes, and a form of metabolic–inflammatory tone influence outcomes, we prospectively evaluated survival machine-learning models, explicitly including hyperuricemia while excluding what we consider major inflammatory confounders. **Methods**: Adults with first-episode unprovoked lower-extremity DVT were enrolled at two centers (July 2024–September 2025). PTS (Villalta) was assessed at 3, 6, 9, and 12 months. The cohort was split 70/30 into training and test sets. Eight learners (RSF, GBM, LASSO + Cox, CoxBoost, survivalsvm, XGBoost-Cox, superpc, and plsRcox) were tuned using 10-fold cross-validation in training and once evaluated in the independent test set. Performance metrics included all time-dependent AUCs, fixed-time ROC AUCs with bootstrap 95% CIs, C-index, various forms of calibration, decision-curve analysis, and simple Kaplan–Meier risk group separation. **Results**: 193 patients were analyzed (PTS in 64%). High 9-month AUCs were seen in training: GBM (0.992) and RSF (0.982) being the strongest; by 12 months, both remained near constant. Test set performance followed a similar pattern, with RSF again favored (AUC 0.948) and XGBoost/GBM close behind. Calibration was satisfactory, net benefit from decision curves positive, and to a large extent, risk groups were separated as expected. **Conclusions**: Survival machine-learning models, at least in this dual-center prospective cohort, produced a clinically useful risk of PTS. Hyperuricemia, or any metabolically based signal, is a valuable addition to the “anatomy and care” of DVT. External validation is still required.

## 1. Introduction

Post-thrombotic syndrome (PTS) is a frequent and clinically consequential complication of lower-extremity deep vein thrombosis (DVT), characterized by chronic pain, edema, skin changes, and functional limitation, with attendant impairment in quality of life and increased health-care utilization [1,2]. Patients with unprovoked DVT are of particular concern because long-term venous sequelae remain difficult to predict early in the clinical course at the point of care [3,4]. Interventions most likely to mitigate PTS—prompt initiation and sustained use of anticoagulation, adherence to compression therapy, and structured follow-up—are time-sensitive and often resource dependent [5]. However, routine practice seldom provides calibrated, horizon-specific estimates of absolute PTS risk, which limits shared decision-making and targeted allocation of preventive care. Accordingly, pragmatic, data-driven tools are needed to translate routinely collected clinical information into individualized estimates of PTS risk during the first year after unprovoked DVT, thereby informing follow-up strategies and supportive interventions.

Venous pathophysiology provides a rationale for prespecifying candidate predictors. Proximal thrombus burden—particularly iliofemoral involvement—may impair venous outflow, damage valves, and sustain venous hypertension; in parallel, care-process variables (time to anticoagulation, duration of anticoagulation, and subsequent use of compression stockings) may modify downstream remodeling [5,6,7]. Beyond these more mechanical and purely procedural factors, we deliberately incorporated a metabolic–inflammatory dimension; hyperuricemia was chosen as the candidate predictor, informed by prior reports linking serum urate to various aspects of endothelial dysfunction, near-constant oxidative stress, and some form of thrombo-inflammatory signaling [8,9]. To reduce nonspecific inflammatory confounding, major inflammatory conditions (e.g., active infection and autoimmune disease) were excluded, while hyperuricemia was retained as the exposure of interest. The model thus captures both actionable care processes and more fundamental thrombus anatomy; testing a readily measurable metabolic marker’s addition of any truly independent prognostic information for post-thrombotic remodeling is the endpoint we are seeking to achieve in unprovoked DVT.

Several limitations in existing prognostic studies for PTS support the need for a revised approach. Many studies are single-center and retrospective, and they often emphasize conventional Cox nomograms with performance assessed at only one time point, thereby providing limited insight into risk trajectories. Reporting of absolute-risk calibration is inconsistent, and clinical utility is seldom quantified using decision-curve methods. Moreover, a clear separation between model development and independent evaluation in a held-out test cohort is absent in some studies, increasing susceptibility to overfitting and optimistic performance estimates [10]. Prior work has variably measured or completely omitted actionable process-of-care factors; metabolic–inflammatory contributors, when addressed, suffer from poor control of confounding. These limitations motivate a prospective design, some form of survival-oriented modeling across multiple horizons, and a more comprehensive evaluation—prediction leading in part to, or at least informing, potential future interventions.

Survival-oriented machine learning offers a suitable framework for this problem. The risk of post-thrombotic syndrome unfolds over time; clinical decisions are inherently horizon-specific. Single-time-point nomograms cannot capture this well, whereas survival learners are capable of estimating risk at multiple, prespecified follow-up points; nonlinearity and various interactions between variables are naturally accommodated within these estimates. A prespecified training–test workflow, using 10-fold cross-validation and a subsequent one-time evaluation against a true held-out test set, helps control overfitting and produces more “deployment-relevant” performance estimates. Comprehensive reporting—time-dependent AUC, some form of fixed-time ROC, concordance, calibration, overall error, and decision-curve analysis—grounds the models’ clinical utility in quantifiable terms. Penalized Cox models provide the near-perfect transparency needed for bedside use; tree/boosting methods, when appropriate, offer the flexible function learning we all seek.

The objective of this study is to develop and compare multiple survival machine-learning models for predicting incident PTS after unprovoked lower-extremity DVT. Routinely available clinical information serves as the basis for all predictions; clinically relevant horizons (3, 6, 9, and 12 months) are specifically targeted for performance quantification. The evaluation emphasizes discrimination, calibration, overall prediction error, and decision value; models generate continuous, patient-level risk estimates, with simple risk groupings used as a secondary output. Model development and subsequent tuning occur entirely within the training set; a single, independent test set appraisal is used for final evaluation, reflecting as closely as possible a form of true prospective use.

This study employs a prospective, dual-center cohort with standardized Villalta assessments at 3/6/9/12 months, a 70/30 training–test split stratified by center and events, and harmonized preprocessing across eight survival learners (penalized Cox, CoxBoost, Random Survival Forest, Gradient Boosting Machine, survivalsvm, XGBoost with a Cox objective, superpc, and plsRcox). The intended contribution is to provide calibrated absolute-risk estimates and actionable summaries that can be integrated into clinical information systems to support tiered follow-up and preventive strategies after unprovoked DVT. External validation and implementation studies remain the necessary next steps for translation of these prediction tools into routine care.

## 2. Materials and Methods

### 2.1. Study Design and Participants

We conducted a prospective, dual-center cohort study at Fuxing Hospital and Xuanwu Hospital (Capital Medical University). The study was conducted in accordance with the Declaration of Helsinki and approved by the Institutional Review Board of Fuxing Hospital, Capital Medical University (protocol code 2025FXHEC-KSP043, approval date 14 January 2025). Adults (≥18 years) with a first episode of unprovoked lower-extremity DVT were enrolled; confirmation of the DVT itself was achieved through either duplex ultrasound or CT venography. In this study cohort, the DVT location was limited to the femoral and iliofemoral veins; isolated distal DVT cases were not included. The “index date” being the date of this diagnosis, participants were enrolled within a prespecified post-diagnosis window. Key exclusions included the following: provoked DVT (post-operative/trauma, pregnancy/puerperium, and/or active cancer), any prior form of PTS, and essential baseline data that exceeded an allowable degree of missingness. Hyperuricemia was a prespecified predictor of interest—reflecting a more systemic inflammatory–metabolic status—and thus conditions capable of confounding various inflammatory profiles were also excluded; active infections, autoimmune diseases, and all chronic non-hyperuricemic inflammatory disorders fell into this category. The study protocol received full approval from the institutional review board (ethics approval 2025FXHEC-KSP043, approval date 14 January 2025); informed consent was obtained from all subjects involved in the study. Standardized enrollment procedures, case ascertainment, and, to a large extent, data capture, were put in place to harmonize definitions across both sites; selection bias remains a low priority but controlled concern.

### 2.2. Outcome, Predictors, Preprocessing, and Data Split

The primary endpoint was incident PTS, adjudicated by trained assessors utilizing the Villalta scale at 3, 6, 9, and 12 months; survival time was defined from the index date to the first occurrence of any form of PTS, with censoring at last contact or end of study. Candidate predictors were pre-specified and clinically motivated; demographics/comorbidities (age, sex, body mass index (BMI), hyperuricemia, and chronic kidney disease (CKD)), thrombus characteristics (notably iliac–femoral involvement), care-process/treatment variables (time-to-anticoagulation, as well as class and duration of anticoagulant use), compression-stocking use/adherence, various lifestyle factors (smoking and alcohol) and baseline laboratories made up the full set. Treatment delay (time to anticoagulant treatment) is defined as the time interval (in days) from the onset of symptoms reported by the patient to the initiation of therapeutic anticoagulant treatment, not the delay after imaging diagnosis. “Anticoagulant type” was defined as the anticoagulant class used at the index presentation (initial treatment phase), whereas the overall duration of anticoagulation was recorded separately as “Anticoag Duration”. Concomitant pulmonary embolism at the index presentation was not prespecified for systematic capture in the study case-report forms and was therefore not included in the current analyses; it was not an exclusion criterion. In the training set, we performed univariable followed by multivariable Cox regression to identify a parsimonious yet clinically plausible subset of predictors. Collinearity was screened, and all subsequent modeling based upon this was reconciled. The dataset was randomly partitioned 70/30 into training and independent test sets, stratified by both center and event type for reproducible analysis. Feature screening and all forms of hyperparameter tuning were kept within the training set; the true, final evaluation of the model was and remains to be held back on the test set.

### 2.3. Model Development

Eight survival learners were trained in the training set using 10-fold cross-validation for tuning/selection, with a fixed random seed (set.seed(123)) across pipelines. For Random Survival Forest (RSF), we used rfsrc with ntree = 1000, nodesize = 3, mtry = 2, splitrule = “logrank”, and enabled importance, proximity, and forest objects. Gradient Boosting Machine (GBM) (gbm, distribution = “coxph”) used n.trees = 1000, interaction.depth = 5, n.minobsinnode = 3, shrinkage = 0.01, and cv.folds = 10 (parallel cores as available). LASSO + Cox employed glmnet (alpha = 1, maxit = 1000), with 10-fold CV (λ_min and λ_1se) for penalty selection consistent with our LASSO CV plots. CoxBoost penalties were optimized via optimCoxBoostPenalty (start.penalty = 200), and the number of boosting steps chosen by 10-fold cv.CoxBoost (maxstepno = 200, type = “verweij”); the final model was refit with stepno = cv.optimal and the selected penalty. For survivalsvm, we used a linear kernel (kernel = “lin_kernel”) with gamma.mu = 0.1, opt.meth = “quadprog”, diff.meth = “makediff3”, sgf.sv = 5, sigf = 7, maxiter = 100, margin = 0.05, bound = 10. XGBoost (Cox) used a tree booster with objective = “survival:cox”, eval_metric = “cox-nloglik”, eta = 0.03, max_depth = 3, subsample = 1, colsample_bytree = 1, gamma = 0.5, nrounds = 100, and early_stopping_rounds = 50. superpc was trained with s0.perc = 0.5 and cross-validated using n.fold = 10, n.threshold = 20, n.components = 3 (with min.features = 5, max.features set to all available, and full CV/prevalidation enabled). For plsRcox, component selection used 10-fold CV up to nt = 10, yielding a final model with nt = 3; risks were then predicted for both training and test sets. To aid interpretability, we examined LASSO coefficient paths and RSF permutation/Gini importance to summarize variable contributions across linear-penalized and tree-based learners. Final models were refit on the full training data using the selected hyperparameters and then evaluated once in the test set without further tuning.

### 2.4. Performance Evaluation and Clinical Utility

Model performance was first assessed within the training set using 10-fold cross-validation, followed by a single evaluation in the independently held-out test set. Accordingly, training-set metrics are presented for the model development context, whereas test-set metrics represent the primary internal validation estimates. Discrimination was quantified through time-dependent AUCs at 3, 6, 9, and 12 months; fixed-time ROC AUCs (also at 9 and 12 months) were used as complements, with bootstrap-derived 95% CIs where applicable. Harrell’s C-index (and its corresponding CI) was reported. Calibration was evaluated both visually and by use, again at regular intervals throughout the follow-up; optimism-correction by some form of bootstrap resampling was applied where possible. Overall prediction error was summarized via Brier and integrated Brier scores. Clinical usefulness was appraised using decision-curve analysis, net benefits compared against “treat-all” and “treat-none”, and upper envelope tracing was the endpoint of interest. Uncertainty around key estimates was characterized by 1000 resamples (two-sided α = 0.05). All pre-specified thresholds, metrics, and/or plotting decisions were made uniformly for both training and test sets.

### 2.5. Risk Stratification, Software, and Reproducibility

For each fitted model, we computed an individual risk score. Risk groups were defined separately within both the training and test cohorts; the cohort-specific median of said score formed the definition of high vs. low groups. Survival functions for these groups were estimated using Kaplan–Meier methods with associated Greenwood standard errors; two-sided log-rank tests served as the means of comparison. Plots were generated via survminer::ggsurvplot with near-identical, consistent options: conf.int = TRUE, pval = TRUE (pval.method was also set to true), surv.median.line = “hv”, legend.labs = c(“high”,”low”), and ggtheme = theme_bw(), a simple two-color palette; risk tables were suppressed. Time origin, censoring rules, and follow-up all matched the primary analysis. All computations took place in R (version 4.5.0); survival, survminer, glmnet, randomForestSRC, CoxBoost, survivalsvm, gbm, xgboost, riskRegression, pec, timeROC, pROC, superpc, and plsRcox, and mice were among the packages used. Reproducibility was a primary concern: set.seed(123) was applied across all pipelines, full hyperparameter grids archived, and/or analysis environments frozen (version controlled). De-identified data and scripts are stored securely on institutional servers to allow for perfect reruns of all analyses.

## 3. Results

### 3.1. Study Cohort and Follow-Up

A total of 249 patients with a first episode of unprovoked lower-extremity DVT were screened. Forty-three patients were excluded according to the predefined exclusion criteria, and 13 patients were lost to follow-up before completion of the 12-month assessment. Therefore, 193 patients were included in the final analysis.

### 3.2. Patient Characteristics and Multivariable Predictors

Baseline characteristics are summarized in Table 1. Patients who developed PTS had lower compression-stocking use and a longer treatment delay (symptom-onset–to–anticoagulation interval), higher BMI, and more prevalent hyperuricemia compared to those without PTS; smoking was the additional more frequent factor, thrombus-location distributions being broadly comparable. Patients who developed PTS were less frequently treated with direct oral anticoagulants (DOACs) and more frequently received low-molecular-weight heparin (LMWH) at the index presentation (Table 1). The training set multivariable Cox model (Table 2) identified iliac–femoral thrombosis, each-day treatment delay, and, to a large extent, hyperuricemia as independent risk factors; anticoagulation for >6 months and compression-stocking use both showed some form of protective effect.

### 3.3. Penalized Selection and Variable Importance

**Figure 1:**
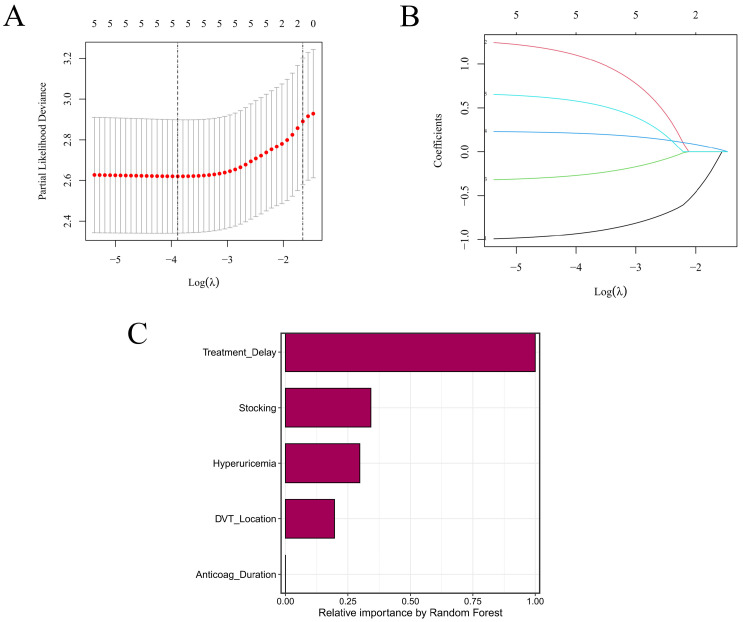
Feature selection and variable importance. (**A**) Ten-fold cross-validated LASSO: mean deviance vs. log(λ), with λ_min and λ_1se marked. (**B**) LASSO coefficient paths across log(λ), highlighting predictors retaining non-zero coefficients near the optimal λ. (**C**) Random Survival Forest permutation importance (VIMP) ranking predictor contributions. Abbreviations: LASSO, least absolute shrinkage and selection operator; VIMP, variable importance.

LASSO with 10-fold cross-validation identified a stable, sparse signature: the error minimum occurred at λ_min, and a comparably performing more parsimonious set was found at λ_1se; coefficient paths retained a core subset (treatment delay, hyperuricemia, iliac–femoral DVT, compression-stocking use, and anticoagulation duration) across all optimal penalties (Figure 1A,B). Concordantly, the Random Survival Forest ranked these same variables among the top contributors on the associated variable-importance plot (Figure 1C).

### 3.4. Discrimination in Training and Test Cohorts

**Figure 2:**
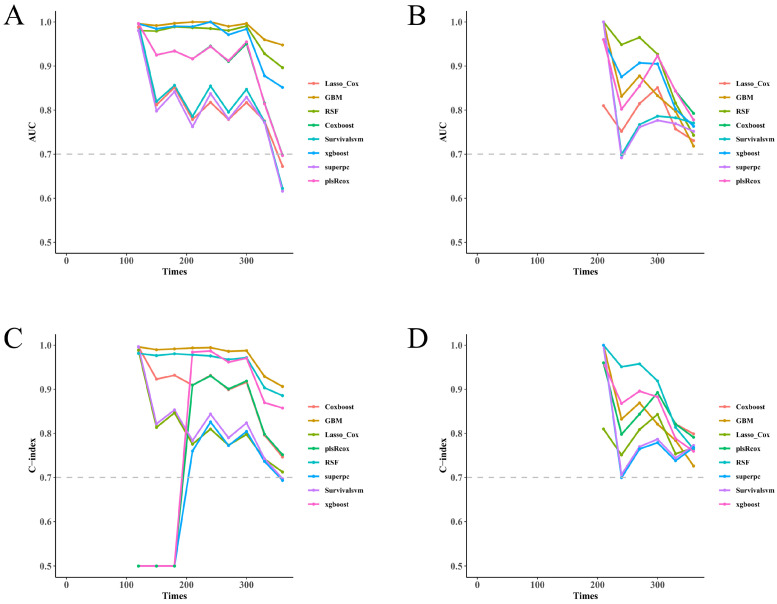
Discrimination performance in training and test cohorts. (**A**) Time-dependent AUC curves in the training set at 3, 6, 9, and 12 months across all models. (**B**) Corresponding curves in the test set. (**C**) Harrell’s C-index in the training set. (**D**) Harrell’s C-index in the test set. Where shown, ribbons/bars denote bootstrap 95% confidence intervals. AUC, area under the time-dependent ROC curve.

Time-dependent AUC curves demonstrated moderate-to-high discrimination within the training set: RSF, GBM, LASSO + Cox, and CoxBoost comprised the “leading tier” of models (Figure 2A). The test set successfully reproduced this relative ordering, with only modest attenuation of the rankings (Figure 2B)—limited overfitting being a key implication of this result. Concordance measures followed suit: stable and to a large extent separable C-index estimates were observed for all models, both in training (Figure 2C) and independent test cohorts (Figure 2D).

### 3.5. Calibration at Prespecified Horizons

**Figure 3:**
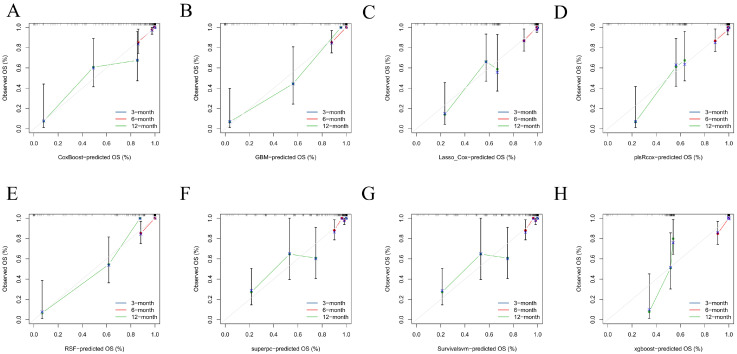
Calibration in the training cohort. Calibration plots comparing predicted versus observed PTS-free survival probability (OS, %) at 3, 6, and 12 months for each model in the training set. (**A**–**H**) correspond to CoxBoost (**A**), GBM (**B**), Lasso-Cox (**C**), plsRcox (**D**), RSF (**E**), superpc (**F**), survivalsvm (**G**), and XGBoost (**H**). The colored curves (blue/red/green) represent the calibration results at 3/6/12 months, respectively, and are drawn by connecting the binned calibration points (shown with marker symbols and vertical uncertainty bars). The grey 45° diagonal line indicates perfect calibration.

**Figure 4:**
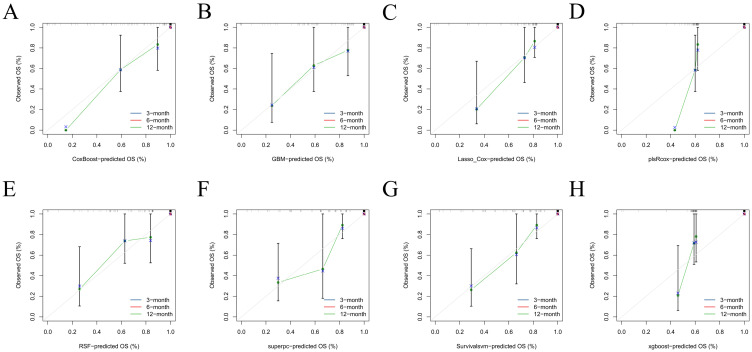
Calibration in the test cohort. Calibration plots comparing predicted versus observed PTS-free survival probability (OS, %) at 3, 6, and 12 months for each model in the independent test set. (**A**–**H**) correspond to CoxBoost (**A**), GBM (**B**), Lasso-Cox (**C**), plsRcox (**D**), RSF (**E**), superpc (**F**), survivalsvm (**G**), and XGBoost (**H**). The colored curves (blue/red/green) represent the calibration results at 3/6/12 months, respectively, and are drawn by connecting the binned calibration points (shown with marker symbols and vertical uncertainty bars). The grey 45° diagonal line indicates perfect calibration.

Calibration plots at 3, 6, and 12 months showed close agreement between predicted and observed risks in the training set (curves tracking the 45° reference with only minor tail deviations; Figure 3) and satisfactory calibration in the test set across all horizons (near-identity behavior through mid-risk with small, clinically modest departures at extremes; Figure 4). Together, these findings indicate good absolute risk calibration at clinically relevant time points.

### 3.6. Clinical Utility by Decision Curve Analysis

**Figure 5:**
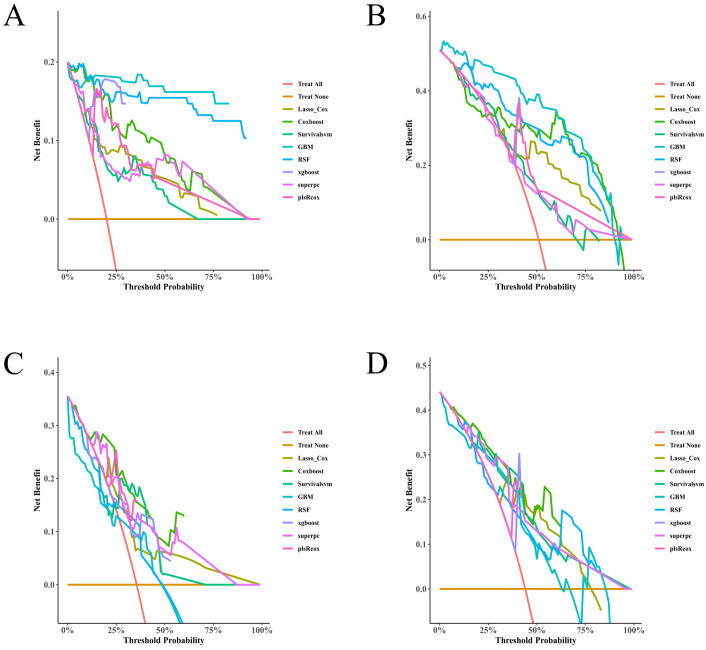
Decision-curve analysis (DCA) at 9 and 12 months. (**A**) Net benefit versus threshold probability at 9 months in the training set. (**B**) Net benefit at 12 months in the training set. (**C**) Net benefit at 9 months in the test set. (**D**) Net benefit at 12 months in the test set. “Treat-all” and “treat-none” strategies are shown for reference; positive net benefit indicates clinical utility over these defaults. DCA quantifies the clinical value of using each model to trigger interventions across threshold probabilities (~0–60%).

Decision curves at month 9 and month 12 demonstrated positive net benefit for the top learners over “treat-all” and “treat-none” across clinically plausible thresholds. In the training set, RSF and GBM typically traced the upper envelope with LASSO + Cox and CoxBoost close behind (Figure 5A,B). Patterns were broadly preserved—though slightly attenuated—in the test set (Figure 5C,D), supporting the clinical decision value of these models at both follow-up points.

### 3.7. Fixed-Time ROC Analysis

**Figure 6:**
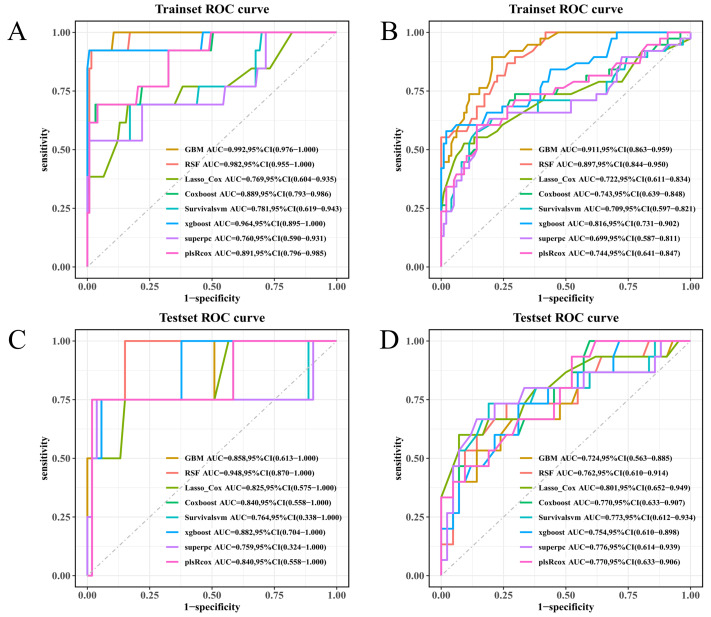
Fixed-time ROC analysis at 9 and 12 months. (**A**) ROC curves at 9 months in the training set with AUC and bootstrap 95% CI reported per model. (**B**) ROC curves at 12 months in the training set. (**C**) ROC curves at 9 months in the test set. (**D**) ROC curves at 12 months in the test set. Higher AUC indicates better discrimination at the specified time horizon.

At 9 months, fixed-time ROC curves within the training set favored GBM/RSF; strong discrimination persisted, if somewhat reduced, by 12 months (Figure 6A,B). The test set showed a more even distribution—RSF led at this early time point, XGBoost being very close behind, all other models converging towards near identical 12-month AUCs; LASSO + Cox was marginally the highest, and the rest were comparable (Figure 6C,D). These same patterns are reflected in the subsequent time-dependent AUC and C-index results.

### 3.8. Risk Stratification Based on Model-Specific Scores

**Figure 7:**
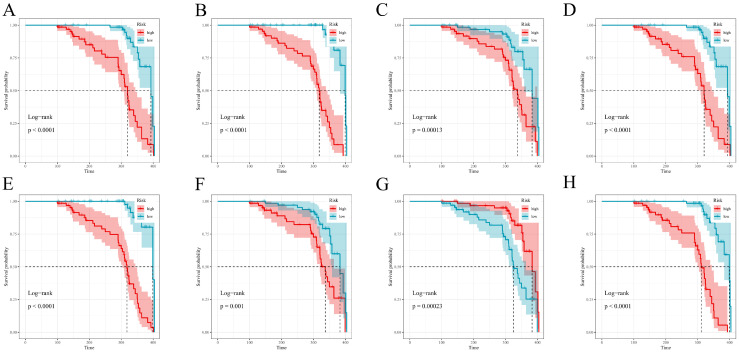
Kaplan–Meier risk stratification in the training cohort. Model-specific high versus low risk groups (defined by the cohort-specific median of each model’s risk score) and PTS-free survival in the training set. (**A**–**H**) correspond to CoxBoost, GBM, LASSO–Cox, plsRcox, RSF, superpc, survivalsvm, and xgboost, respectively. Shaded areas (where shown) denote 95% CIs; *p*-values are from two-sided log-rank tests. Time origin and censoring match the primary analysis.

**Figure 8:**
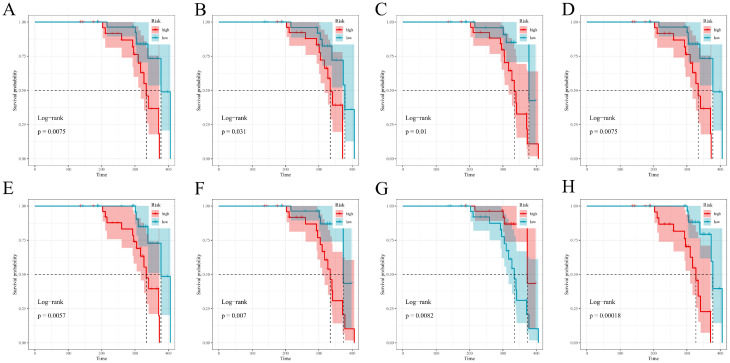
Kaplan–Meier risk stratification in the test cohort. Model-specific high versus low risk groups (defined by the cohort-specific median of each model’s risk score) and PTS-free survival in the test set. (**A**–**H**) correspond to CoxBoost, GBM, LASSO–Cox, plsRcox, RSF, superpc, survivalsvm, and xgboost, respectively. Risk groups are defined by the median model-specific score within the test cohort; log-rank *p*-values assess high/low separation.

Model-derived risk scores produced clear high- vs. low-risk separation on Kaplan–Meier curves throughout follow-up in the training set; GBM and RSF showed the most prominent separation, strong (though less obvious) separation being observed for CoxBoost and XGBoost; LASSO–Cox, survivalsvm, plsRcox, and superpc all exhibited significant but comparatively narrower gaps (Figure 7A–H). The test set confirmed generalizable stratification across all eight models, with XGBoost and, to a large extent, RSF retaining the widest possible survival gaps; the other models showed more or less consistent clinically meaningful divergence (Figure 8A–H).

## 4. Discussion

In this prospective, dual-center cohort of unprovoked lower-extremity DVT, multiple survival machine-learning models demonstrated consistent predictive performance and potential clinical utility. Discrimination was moderate to high in both the training and test cohorts as assessed by time-dependent AUC, and the relative ranking of models was broadly preserved (Figure 2A–D), with fixed-time ROC analyses at 9 and 12 months providing concordant results (Figure 6A–D). Predicted absolute risks corresponded well with observed outcomes at the prespecified horizons of 3, 6, and 12 months, indicating satisfactory calibration in both cohorts (Figure 3 and Figure 4). Decision-curve analysis suggested positive net benefit for the top-performing models across clinically plausible thresholds, supporting the concept that model-based risk estimates could inform follow-up intensity and supportive care (Figure 5A–D). Risk scores derived from each model stratified patients into distinct PTS-free survival groups over follow-up (Figure 7 and Figure 8). Overall, the prespecified train/test separation, 10-fold cross-validation within training, and multi-metric evaluation support reproducibility and provide a foundation for future external validation.

Prior prognostic studies of PTS have largely been retrospective and single-center, often relying on conventional Cox nomograms evaluated at a single horizon; absolute-risk calibration and explicit assessments of clinical utility have received comparatively limited attention. Across this study, proximal thrombus burden, delayed or suboptimal anticoagulation, and inconsistent use of compression stockings have been repeatedly reported as relevant factors [11,12,13,14]. Effect sizes and “transportability” of these factors, however, show considerable variation—heterogeneity in case mix, endpoint definitions, and, to a large extent, follow-up windows are believed to be responsible. Using a prospective dual-center cohort, strict 10-fold training/test set separation, multi-horizon performance reporting, and decision-curve net benefit as a form of clinical utility metric, our study extends this line of work towards truly deployable predictive tools. A key emphasis was linking prediction to partially modifiable care processes assessed at clinically relevant time points (3, 6, and 12 months), which have been inconsistently handled in prior models.

The association of iliac–femoral thrombosis with a higher PTS risk is biologically consistent: thrombus within this specific segment implies a greater clot burden and, by extension, a more persistent form of outflow obstruction [7,14]. Valve injury and chronic venous hypertension are other direct consequences of such a state—all being central to the established pathophysiology of PTS10. This predictor’s persistence following multivariable adjustment suggests it captures some form of structural substrate; demographic and treatment-related factors cannot fully account for this effect. Time to anticoagulation emerged as another very robust determinant. Earlier initiation plausibly limits propagation of the thrombus, promotes recanalization in at least some cases, and reduces various types of inflammatory damage to (near-)normal venous valves; daily delays in starting anticoagulation represent an increasing window for organization and associated fibrosis [15,16]. From a care-delivery perspective, streamlining diagnostic pathways, timely initiation of anticoagulation, and standardized discharge processes may therefore represent actionable targets to mitigate downstream PTS risk.

To reduce inflammatory confounding, we excluded active infection, autoimmune disease, and chronic inflammatory disorders other than hyperuricemia; within this restricted background, an association with hyperuricemia can be interpreted in the context of inflammatory mechanisms relevant to venous disease. Initiation and propagation of DVT involve inflammatory pathways such as endothelial activation, leukocyte adhesion, monocyte tissue-factor expression, and NETosis, which promote thrombin generation and contribute to thrombus stabilization; systemic low-grade inflammation may prime both platelets and the endothelium [17,18,19]. Hyperuricemia amplifies these processes: xanthine oxidase-derived reactive oxygen species, reduced nitric-oxide bioavailability, and, to a larger extent, NLRP3 inflammasome activation (IL-1β/IL-18) allow for near-constant “inflammatory priming” [20]. Upregulation of adhesion molecules and/or impaired endothelial repair are additional consequences. The post-thrombotic phase sees persistent venous hypertension driving a form of sterile inflammation; vein-wall remodeling (leukocyte infiltration, MMPs, collagen deposition, and valve damage) is the end result—all slowed, if not completely inhibited, by the ongoing pro-inflammatory and/or oxidative stress milieus [21,22]. Thus, hyperuricemia may function as a tractable marker of a hostile healing environment; recanalization and/or PTS risk are both affected by this. Clinically, routine urate profiling in DVT pathways at least partially addresses risk conversations. Weight management, diet, and/or future urate-lowering therapy should be considered. Causality remains to be seen, but improving venous healing is a real and important clinical outcome [23,24].

Consistent with venous-rehabilitation principles, compression-stocking use showed a protective association. Beyond simple edema control, effective compression reduces venous hypertension and the associated shear-induced inflammation; limiting the subsequent cascade towards fibrosis and various forms of valve incompetence is a key benefit. Adherence drives this benefit, and therefore our results call for some sort of structured adherence support (education, fitting, follow-up troubleshooting) rather than just a prescription of the stockings themselves [25]. Anticoagulation > 6 months was similarly associated with lower PTS risk within our cohort when compared to shorter courses. Primarily aimed at recurrent thromboembolism, adequate duration of anticoagulation may also promote a more stable recanalization—late extension of thrombus being one of the worst-case scenarios compromising valve integrity [6,26]. Decisions regarding anticoagulation duration must continue to balance bleeding risk against competing indications; nevertheless, the observed associations support individualized discussion of time-dependent management factors. Collectively, timely anticoagulation, adherence to compression therapy, and other modifiable care-process factors identified by prediction models may help inform patient-level follow-up and supportive strategies.

For real-world translation, the risk model could be integrated into existing clinical information systems (for example, an electronic health record or some form of institutional electronic medical record). Implementation should (i) map the required predictors to routinely captured fields (thrombus segment, time to anticoagulation, urate level, and compression-stocking use being prime examples), (ii) compute a continuous risk estimate at prespecified follow-up time points and display this alongside brief, action-oriented guidance regarding follow-up intensity, and (iii) permit periodic monitoring of both calibration drift and various outcome-linked quality indicators (adherence, revisit rates, patient-reported symptoms). Computational resources or transparency at the point of care being top priorities would lead to a parsimonious version of the model being used; tree-based implementations, should they offer any meaningful performance gain, could remain as a back-end service. Performance is not the end goal, but all forms of implementation should at least partially answer the clinical question the model was designed to address.

This study has several strengths. It used a prospective, dual-center cohort of unprovoked DVT with harmonized recruitment procedures and standardized Villalta assessments at 3, 6, 9, and 12 months. Model development followed a prespecified 70/30 training–test split, with 10-fold cross-validation restricted to the training set and a single final evaluation in the held-out test set. Multiple survival learners were benchmarked using consistent preprocessing and candidate-predictor handling, and performance was assessed using prespecified metrics, including discrimination, calibration, overall prediction error, and decision value. Reproducibility was supported through fixed random seeds, archived parameter grids, and a stable analysis environment. Finally, the exclusion of active infection, autoimmune disease, and most other chronic inflammatory conditions (while retaining hyperuricemia as the exposure of interest) reduced major inflammatory confounding and enabled a more targeted assessment of a metabolically related signal.

Several limitations merit consideration. This prospective observational cohort study was not prospectively registered on a public clinical trial registry platform. This cohort comprised proximal (femoral or iliofemoral) unprovoked DVT only; isolated distal DVT was not represented, which limits extrapolation of our findings to patients with distal DVT. Furthermore, because hyperuricemia was a prespecified predictor of interest, we excluded conditions that could substantially confound inflammatory profiles (e.g., active infection, autoimmune disease, and chronic non-hyperuricemic inflammatory disorders); therefore, our results may be less generalizable to broader unprovoked DVT populations with concurrent inflammatory comorbidity. Sample size and subsequent event counts constrain precision at some horizons, particularly within subgroups; wider confidence intervals are a direct consequence. Both centers are part of the same academic system, meaning transportability must be externally validated. Targeted exclusions were used, but residual confounding likely persists; adherence and/or timing variables were captured in a pragmatic manner. Kaplan–Meier risk stratification used median splits for communication; these groupings should not be interpreted as defining optimal intervention thresholds. In addition, imaging-derived quantification of thrombus burden, longitudinal inflammatory biomarkers, and formal modeling of bleeding-risk trade-offs were not incorporated. Finally, the study design is predictive rather than causal, and future work could explore extensions such as competing-risk approaches.

## 5. Conclusions

In this prospective two-center cohort of patients with proximal unprovoked lower-extremity DVT, higher baseline serum uric acid (hyperuricemia) was associated with an increased risk of PTS during follow-up. In this selected population, hyperuricemia may serve as an additional, readily available risk factor to refine PTS risk stratification alongside established clinical predictors.

## Figures and Tables

**Table 1:** Characteristics of included patients.

Demographic Characteristics	Levels	No Post-Thrombotic Syndrome (*N* = 129)	Post-Thrombotic Syndrome (*N* = 64)	*p*-Value
Gender	Female	72 (55.8%)	33 (51.6%)	0.686
	Male	57 (44.2%)	31 (48.4%)	
Stocking	No	32 (24.8%)	37 (57.8%)	<0.001
	Yes	97 (75.2%)	27 (42.2%)	
Smoking	No	79 (61.2%)	24 (37.5%)	0.003
	Yes	50 (38.8%)	40 (62.5%)	
Alcohol	No	84 (65.1%)	54 (84.4%)	0.009
	Yes	45 (34.9%)	10 (15.6%)	
DVT Location	Femoral vein thrombosis	84 (65.1%)	43 (67.2%)	0.901
	Iliofemoral vein thrombosis	45 (34.9%)	21 (32.8%)	
Hypertension	No	65 (50.4%)	36 (56.2%)	0.539
	Yes	64 (49.6%)	28 (43.8%)	
Diabetes	No	80 (62%)	42 (65.6%)	0.741
	Yes	49 (38%)	22 (34.4%)	
CHD	No	81 (62.8%)	39 (60.9%)	0.926
	Yes	48 (37.2%)	25 (39.1%)	
CKD	No	119 (92.2%)	22 (34.4%)	<0.001
	Yes	10 (7.8%)	42 (65.6%)	
Anticoag Duration	<3 m	33 (25.6%)	27 (42.2%)	0.018
	>6 m	57 (44.2%)	16 (25%)	
	3–6 m	39 (30.2%)	21 (32.8%)	
Thrombectomy	No	89 (69%)	46 (71.9%)	0.807
	Yes	40 (31%)	18 (28.1%)	
Hyperlipidemia	No	61 (47.3%)	33 (51.6%)	0.684
	Yes	68 (52.7%)	31 (48.4%)	
Statin	No	43 (33.3%)	29 (45.3%)	0.144
	Yes	86 (66.7%)	35 (54.7%)	
Antiplatelet	No	82 (63.6%)	38 (59.4%)	0.684
	Yes	47 (36.4%)	26 (40.6%)	
Anticoagulant Type	DOACs	55 (42.6%)	22 (34.4%)	0.038
	LMWH	10 (7.8%)	13 (20.3%)	
	VKA	64 (49.6%)	29 (45.3%)	
Treatment Delay	Mean ± SD	6.7 ± 2.9	9.2 ± 4.0	<0.001
Age	Mean ± SD	61.7 ± 5.8	63.4 ± 6.1	0.06
Hyperuricemia	No	118 (91.5%)	43 (67.2%)	<0.001
	Yes	11 (8.5%)	21 (32.8%)	
BMI	Mean ± SD	21.6 ± 2.1	25.3 ± 1.6	<0.001

**Table 2:** Training set univariate and multivariate COX regression results.

Variable Name	Desc	Stats	HR (Univariable)	HR (Multivariable)
Gender	Female	70 (51.5%)		
	Male	66 (48.5%)	1.12 (0.60–2.06, *p* = 0.727)	
Stocking	No	49 (36.0%)		
	Yes	87 (64.0%)	0.24 (0.13–0.45, *p* < 0.001)	0.36 (0.17–0.78, *p* = 0.010)
Smoking	No	73 (53.7%)		
	Yes	63 (46.3%)	1.55 (0.83–2.89, *p* = 0.172)	
Alcohol	No	99 (72.8%)		
	Yes	37 (27.2%)	0.72 (0.33–1.57, *p* = 0.413)	
DVT Location	Femoral vein thrombosis	89 (65.4%)		
	Iliofemoral vein thrombosis	47 (34.6%)	4.32 (2.15–8.68, *p* < 0.001)	3.06 (1.28–7.34, *p* = 0.012)
Hypertension	No	73 (53.7%)		
	Yes	63 (46.3%)	1.26 (0.68–2.34, *p* = 0.465)	
Diabetes	No	79 (58.1%)		
	Yes	57 (41.9%)	0.87 (0.47–1.62, *p* = 0.661)	
CHD	No	88 (64.7%)		
	Yes	48 (35.3%)	0.82 (0.43–1.56, *p* = 0.555)	
CKD	No	103 (75.7%)		
	Yes	33 (24.3%)	3.23 (1.72–6.08, *p* < 0.001)	1.54 (0.72–3.25, *p* = 0.263)
Anticoag Duration	<3 m	38 (28.0%)		
	3–6 m	43 (31.6%)	2.37 (1.09–5.13, *p* = 0.029)	1.31 (0.51–3.34, *p* = 0.574)
	>6 m	55 (40.4%)	0.43 (0.20–0.93, *p* = 0.032)	0.42 (0.18–0.96, *p* = 0.039)
Thrombectomy	No	97 (71.3%)		
	Yes	39 (28.7%)	0.67 (0.32–1.40, *p* = 0.285)	
Hyperlipidemia	No	64 (47.1%)		
	Yes	72 (52.9%)	1.03 (0.56–1.89, *p* = 0.921)	
Statin	No	48 (35.3%)		
	Yes	88 (64.7%)	1.00 (0.55–1.83, *p* = 0.998)	
Antiplatelet	No	87 (64.0%)		
	Yes	49 (36.0%)	1.26 (0.69–2.32, *p* = 0.453)	
Anticoagulant Type	LMWH	21 (15.4%)		
	DOACs	56 (41.2%)	0.19 (0.08–0.43, *p* < 0.001)	0.63 (0.22–1.78, *p* = 0.382)
	VKA	59 (43.4%)	0.42 (0.20–0.87, *p* = 0.020)	1.42 (0.57–3.53, *p* = 0.455)
Treatment Delay	Mean ± SD	7.6 ± 3.3	1.26 (1.15–1.39, *p* < 0.001)	1.40 (1.22–1.61, *p* < 0.001)
Age	Mean ± SD	62.7 ± 6.1	1.05 (0.99–1.10, *p* = 0.087)	
Hyperuricemia	No	112 (82.4%)		
	Yes	24 (17.6%)	2.08 (1.09–3.96, *p* = 0.026)	3.55 (1.52–8.32, *p* = 0.004)
BMI	Mean ± SD	23.0 ± 2.6	1.43 (1.25–1.64, *p* < 0.001)	1.15 (0.96–1.37, *p* = 0.129)

## Data Availability

The data presented in this study are available on request from the corresponding author due to reasonable request.

## Abbreviations

The following abbreviations are used in this manuscript:

PTS	Post-thrombotic syndrome
DVT	deep vein thrombosis
CKD	chronic kidney disease
BMI	body mass index
DOAC	direct oral anticoagulant
LMWH	low-molecular-weight heparin
RSF	Random Survival Forest
GBM	Gradient Boosting Machine

## References

[1] Donbaloğlu M.O., Gürkan S., Gür Ö. (2024). Do treatment methods for deep vein thrombosis have different effects on post-thrombotic syndrome and the quality of life?. Vascular.

[2] Silva J.C., Constâncio V., Lima P., Nunes C., Silva E., Anacleto G., Fonseca M. (2022). Determinants of Quality of Life in Patients with Post-Thrombotic Syndrome. Ann. Vasc. Surg..

[3] Prandoni P., Ageno W., Ciammaichella M., Mumoli N., Zanatta N., Imberti D., Visonà A., Bucherini E., Di Nisio M., DOAC-PTS Investigators (2020). The risk of post-thrombotic syndrome in patients with proximal deep vein thrombosis treated with the direct oral anticoagulants. Intern. Emerg. Med..

[4] Kahn S.R., Comerota A.J., Cushman M., Evans N.S., Ginsberg J.S., Goldenberg N.A., Gupta D.K., Prandoni P., Vedantham S., Walsh M.E. (2014). The postthrombotic syndrome: Evidence-based prevention, diagnosis, and treatment strategies: A scientific statement from the American Heart Association. Circulation.

[5] Nielsen J.D., Hermann T.S., Fredskilde P.C.A. (2024). Graduated elastic compression stockings in the prevention of post-thrombotic syndrome: A systematic review and meta-analysis. Phlebology.

[6] Guzel A., Canbaz S. (2025). A retrospective assessment of venous recanalization outcomes for oral anticoagulant treatment in deep vein thrombosis. Vascular.

[7] Li W., Vedantham S., Jaffer F.A., Kakkos S.K., Galanaud J.-P., Dobesh P.P., Fukaya E., Whipple M.O., Alabi O., Rosovsky R.P. (2025). Revisiting the Open Vein Hypothesis to Reduce the Postthrombotic Syndrome: Implications for Multidisciplinary Care and Research: A Scientific Statement From the American Heart Association. Circulation.

[8] Baaten C., Vondenhoff S., Noels H. (2023). Endothelial Cell Dysfunction and Increased Cardiovascular Risk in Patients With Chronic Kidney Disease. Circ. Res..

[9] Li H., Huang J., Sun M. (2025). The relationship among inflammatory biomarkers, hyperuricemia and chronic kidney disease: Analysis of the NHANES 2015–2020. Ren. Fail..

[10] Chaitidis N., Kokkinidis D.G., Papadopoulou Z., Hasemaki N., Attaran R., Bakoyiannis C. (2022). Management of Post-thrombotic Syndrome: A Comprehensive Review. Curr. Pharm. Des..

[11] Guo X., Xu H., Zhang J., Hao B., Yang T. (2023). A systematic review and meta-analysis of risk prediction models for post-thrombotic syndrome in patients with deep vein thrombosis. Heliyon.

[12] Mulatu A., Melaku T., Chelkeba L. (2020). Deep Venous Thrombosis Recurrence and Its Predictors at Selected Tertiary Hospitals in Ethiopia: A Prospective Cohort Study. Clin. Appl. Thromb. Hemost..

[13] Serhal M., Barnes G.D. (2019). Venous thromboembolism: A clinician update. Vasc. Med..

[14] Spiezia L., Campello E., Simion C., Poretto A., Dalla Valle F., Simioni P. (2022). Risk Factors for Post-Thrombotic Syndrome in Patients With a First Proximal Deep Venous Thrombosis Treated With Direct Oral Anticoagulants. Angiology.

[15] Singh S., Kumar P., Yadav S.K., Jaffer F.A., Reed G.L. (2025). Recent Pathophysiological Insights Are Advancing the Treatment of Venous Thromboembolism. JACC Basic. Transl. Sci..

[16] Tang Y., Luo Z., Ye X. (2025). Isolated distal deep vein thrombosis: Diagnosis and management. Sci. Prog..

[17] Colling M.E., Tourdot B.E., Kanthi Y. (2021). Inflammation, Infection and Venous Thromboembolism. Circ. Res..

[18] Varjú I., Tanka-Salamon A., Kolev K. (2025). Neutrophil Extracellular Traps: At the Interface of Thrombosis and Comorbidities. Semin. Thromb. Hemost..

[19] Zhang Z., Hu J., Bai Y., Liang W., Jin Y. (2025). Emerging molecular targets in deep vein thrombosis: From inflammation to coagulation. Hematology.

[20] An M.F., Wang M.Y., Shen C., Sun Z.-R., Zhao Y.-L., Wang X.-J., Sheng J. (2021). Isoorientin exerts a urate-lowering effect through inhibition of xanthine oxidase and regulation of the TLR4-NLRP3 inflammasome signaling pathway. J. Nat. Med..

[21] Fang F., Yang H., Wang X., Zhao T., Zhao P., Liu X. (2025). Extracellular Vesicles in Atherosclerosis: From Pathogenesis to Theranostic Applications. Small.

[22] Shao F., He R., Jin Y., Pang Y., Sun Z., Zhang B., Jin S. (2025). Regulation and Clinical Application of Exosomes in Venous Thrombosis. Angiology.

[23] De Lucchi L., Nardin C., Sponchiado A., Raggi D., Faggin E., Martini E., Pagliara V., Callegari E., Caberlotto L., Plebani M. (2021). Serum uric acid levels and the risk of recurrent venous thromboembolism. J. Thromb. Haemost..

[24] Weng H., Li H., Zhang Z., Zhang Y., Xi L., Zhang D., Deng C., Wang D., Chen R., Chen G. (2023). Association between uric acid and risk of venous thromboembolism in East Asian populations: A cohort and Mendelian randomization study. Lancet Reg. Health West. Pac..

[25] Amin E., Joore M.A., ten Cate-Hoek A.J. (2016). Compression to prevent PTS: A controversy?. Phlebology.

[26] Rennenberg R.J. (2016). Oral anticoagulants, effect on thrombus resolution and post-thrombotic syndrome. Phlebology.

